# Calvarial Plasmacytoma Mimicking Meningioma as the Initial Presentation of Multiple Myeloma

**DOI:** 10.7759/cureus.1126

**Published:** 2017-03-29

**Authors:** Peter Morgenstern, David Pisapia, Rohan Ramakrishna

**Affiliations:** 1 Department of Neurological Surgery, New York-Presbyterian/Weill Cornell Medical Center; 2 Pathology, Weill Cornell Medical College; 3 Department of Neurological Surgery, Weill Cornell Medical College

**Keywords:** meningioma, plasmacytoma, multiple myeloma

## Abstract

Plasmacytoma of the calvarium is a well-described feature of multiple myeloma and in some cases has been reported as a solitary lesion. However, when associated with multiple myeloma these are typically identified after the initial diagnosis is made. This case is unusual in that the diagnosis of plasmacytoma was first suspected in a patient thought to have a meningioma on the day of surgery, when a magnetic resonance imaging (MRI) demonstrated spontaneous involution of the mass. Recognition of evolving changes in a calvarial or dural-based lesion should prompt the practitioner to consider alternative diagnoses other than meningioma prior to proceeding with surgical resection.

## Introduction

Plasma cell tumors are monoclonal proliferations of plasma cells that are most frequently observed in the context of their disseminated form: multiple myeloma. However, they may also occur as solitary plasmacytomas. Cranial plasmacytomas may arise from the skull, leptomeninges, or the brain parenchyma [1]. When observed without an existing diagnosis of multiple myeloma, these tumors can easily be mistaken for meningioma, metastasis, or lymphoma. Because of its high prevalence, meningioma is the most common misdiagnosis among patients with plasmacytoma [1]. We present a case in which a patient was thought to have a meningioma in the clinic and scheduled for surgical resection. At the time of surgery he was found to have a plasmacytoma and later diagnosed with multiple myeloma.

## Case presentation

A 57-year-old man presented to the neurosurgical clinic complaining of headache after a workplace accident. He was found to have a right calvarial mass with compression of the right frontal lobe on head computed tomography (CT). A magnetic resonance imaging (MRI) of the brain demonstrated a contrast enhancing calvarial mass with features consistent with meningioma. In the context of the patient’s unremarkable medical history, poorly controlled headaches and age, he was scheduled for elective resection. Aside from acetaminophen he took no additional medications for pain and was not started on any anti-inflammatory or steroid medications.

**Figure 1:**
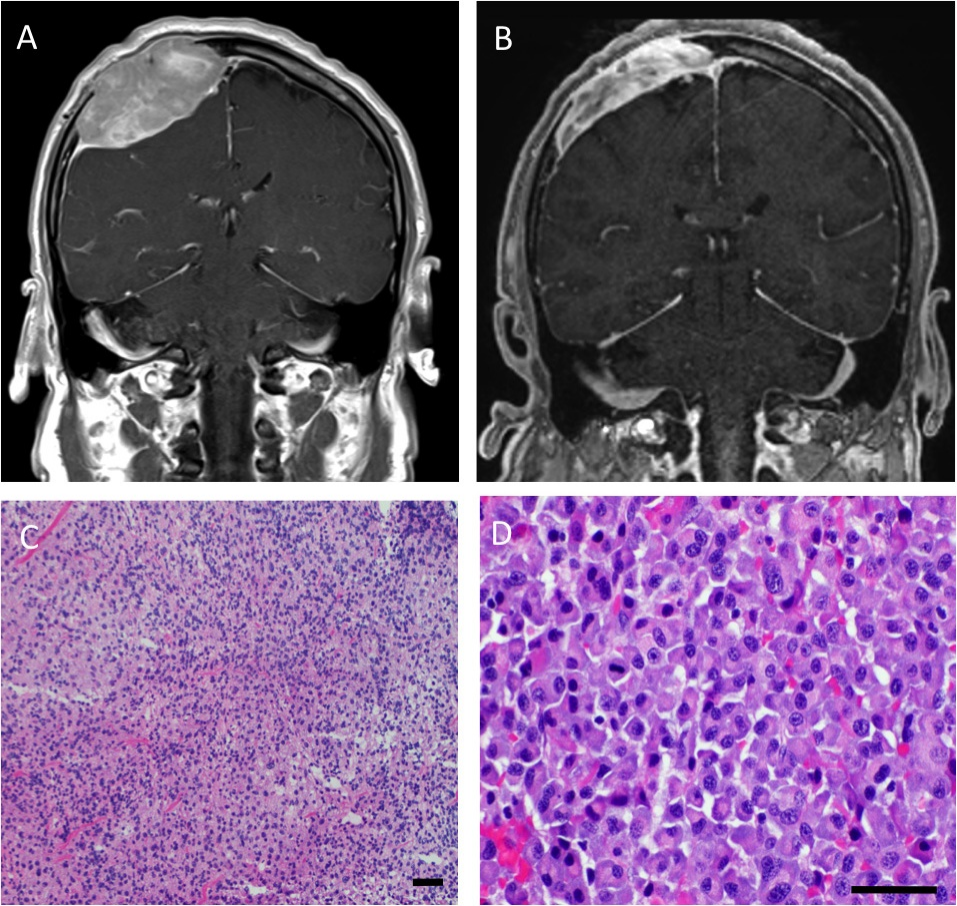
Radiographic and histopathologic images Coronal gadolinium enhanced T1-weighted magnetic resonance imaging (MRI) at diagnosis (A) and on the day of surgery (B) demonstrates reduction in the size of the right calvarial lesion. Hematoxylin and eosin staining of the frozen section (C) and permanent section (D, higher power) shows more distinct plasmacytoid morphology in the absence of frozen section artifact. Permanent section at high power reveals atypical plasma cells with mitotic figures, and multinucleation. Scale bars = 50 μm.

An MRI obtained three weeks later on the morning of surgery for use with intraoperative neuronavigation demonstrated a spontaneous reduction in the size of the lesion, raising the suspicion of an alternative diagnosis to meningioma (Figure 1). In discussion with the patient it was decided that, because of the size of the lesion and associated mass effect, it would be prudent to proceed with a minimally invasive biopsy followed by resection depending on the preliminary pathologic diagnosis. A frozen section at the time of surgery was non-diagnostic and described as a cellular neoplasm that could be consistent with an atypical meningioma given the sheet-like architecture. Lymphoma was not suspected given the cell size and amount of cytoplasm, and a plasmacytoid morphology was not appreciated, hampered in part by frozen section artifact. A gross total resection was performed. The patient recovered well and had an unremarkable perioperative course.

After permanent processing, the tumor was identified as a plasmacytoma (CD138^+^, CD56^+^, Lambda^+^, Kappa^-^) with overall 30% Ki67 positivity, eight percent in the CD138^+^ population. The patient underwent a systemic evaluation postoperatively, which revealed multiple lytic lesions in the humerus, glenoid process, and ribs. Serum protein electrophoresis (SPEP) was positive for immunoglobulin G (IgG)-Lambda, establishing the diagnosis of multiple myeloma. He initiated therapy in consultation with his hematologist/oncologist during the postoperative period. At nine months follow-up, he was doing well with a Karnofsky performance status (KPS) of 90 and continuing maintenance chemotherapy.

## Discussion

Dural-based plasmacytoma mimicking meningioma has been described previously, typically involving the leptomeninges and skull concurrently [1-5]. Solitary cranial plasmacytoma occurs rarely and usually occurs as a manifestation of multiple myeloma as in this case [2-4,6]. However, spontaneous involution of a plasmacytoma without therapeutic intervention has not been previously reported.

Because of their rarity, the literature on the management of cranial plasmacytoma is limited. One important facet of this discussion is the role of surgical resection. Consensus at this time suggests that for solitary plasmacytoma the management is gross total resection with adjuvant radiation therapy [1-2,7-8]. However, the appropriate management of plasmacytomas associated with multiple myeloma is less definitive. Most authors suggest chemotherapy with radiation as the first line for these lesions, even when there is a mass effect, because of their exquisite sensitivity to this approach [9-10]. Surgical resection is typically reserved for those refractory to therapy or unable to receive radiation due to proximity to sensitive structures such as the optic chiasm. A histopathologic study by Schwartz, et al. examined cell adhesion molecules and plasmacytoma location and a possible correlation to later development of multiple myeloma. It was suggested that cranial base location is more predictive of a subsequent diagnosis of multiple myeloma. Furthermore, it was noted that extramedullary dural-based lesions were unlikely to be associated with multiple myeloma and therefore should be resected [2]. Our case is an exception to this conclusion in that a convexity dural-based lesion represented a plasmacytoma that was later found to be a presentation of multiple myeloma.

In the case described here, the differential diagnosis at the time of intraoperative frozen section included meningioma and solitary plasmacytoma, and gross total resection was performed. However, given the subsequent diagnosis of multiple myeloma in the first several days postoperatively, we would recommend a preoperative workup for multiple myeloma prior to making treatment decisions when plasmacytoma enters the differential diagnosis.

## Conclusions

This case is unusual in that a tumor initially thought to be meningioma reduced in size substantially prior to surgery in the absence of steroid or other medical therapy. Identification of this finding preoperatively altered the initial operative plan, prompting a small preliminary biopsy prior to proceeding with resection. Recognition of such evolution of a dural-based or calvarial mass should prompt further evaluation prior to proceeding with surgery in order to identify occult cases of multiple myeloma.
